# Combined Prosthesis with Extracoronal Castable Precision Attachments

**DOI:** 10.1155/2013/282617

**Published:** 2013-12-09

**Authors:** Naveen Gupta, Abhilasha Bhasin, Parul Gupta, Pankaj Malhotra

**Affiliations:** ^1^Department of Prosthodontics, Institute of Dental Education and Advanced Studies (IDEAS), Gwalior, India; ^2^Department of Prosthodontics, Hitkarini Dental College and Hospital, Jabalpur, India; ^3^Smile N Care Dental Clinics, New Delhi, India; ^4^Department of Periodontics, Institute of Dental Education and Advanced Studies, Gwalior, MP, India

## Abstract

Satisfactory restoration in a patient with a partially edentulous situation can be challenging especially when unilateral or bilateral posterior segment of teeth is missing. Successful restoration can be done with various conventional and contemporary treatment options. One such treatment modality is attachment-retained cast partial dentures. This paper describes a case report of a patient with maxillary bilateral distal extension edentulous span restored with a cast partial denture having an extracoronal castable precision attachment (RHEIN 83 OT CAP attachments system).

## 1. Introduction 

Esthetically and functionally successful prosthetic rehabilitation requires careful attention and meticulous treatment planning. Rehabilitation of partially edentulous arch can be challenging when it is a distal extention situation classified under Kennedy's class I and class II situations [1]. In such a condition, a fixed partial denture cannot be fabricated because of missing distal abutment. Implant-supported prosthesis can be planned, but it is sometimes not feasible due to insufficient amount of bone and economic reason. So, in such situation an acrylic partial denture or a cast partial denture is largely preferred. Cast partial dentures are made retentive by the use of retainers and precision attachment components [2]. Precision attachments could be extracoronal and intracoronal. Attachment-retained cast partial dentures facilitate both esthetic and functional replacement of missing teeth. Studies have shown a survival rate of 83.35% for 5 years, of 67.3% up to 15 years, and of 50% when extrapolated to 20 years [3, 4]. This paper describes a case report of a patient with maxillary bilateral distal extension Kennedy's class I condition which is prosthetically restored by a cast partial denture retained using a extracoronal castable precision attachment (RHEIN 83 OT CAP attachments system).

## 2. Case Report 

**Figure 1:**
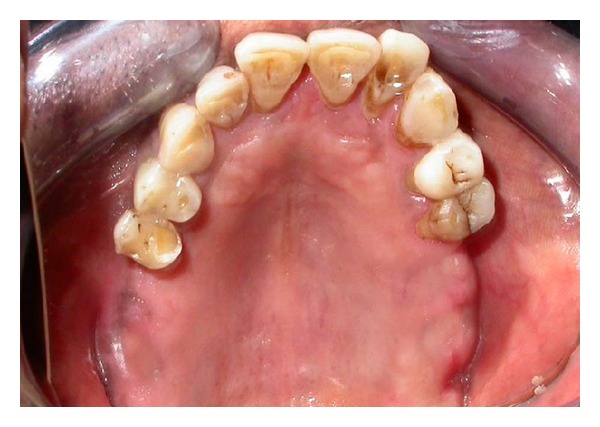
Maxillary arch showing Kennedy's class 1 situation.

A 50-year-old male reported with missing maxillary molar teeth bilaterally. He gave a history of unsatisfactory acrylic partial denture wearing. On intraoral examination, it was noted that the patient had missing maxillary I, II, and III molars bilaterally (Kennedy's class I) and completely edentulous mandibular arch. The remaining teeth in maxillary arch were periodontally stable (Figure 1).

**Figure 2:**
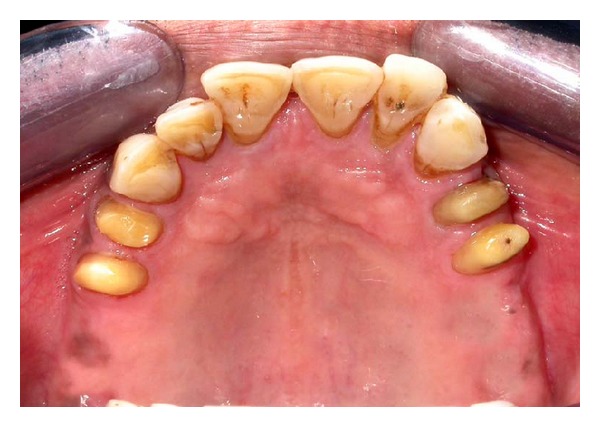
Teeth numbers 14, 14, 24, and 25 prepared to receive porcelain fused to metal crowns.

After complete clinical and radiographic examination, a prosthetic treatment plan was set up. Combined prosthesis with extracoronal precision attachment was planned for maxillary bilateral distal extension arch and complete denture for mandibular arch. Tooth preparation of numbers 14, 15, 24, and 25 abutment teeth was performed to receive porcelain fused to metal crowns (Figure 2). The abutments prepared were temporized after making definitive impression.

### 2.1. Lab Procedure

Waxing up of abutments 14, 15, 24, and 25 was performed and milling of lingual area of metal ceramic setup was done. Articulation spaces and bulkiness were evaluated in order to proceed with optimal positioning of attachments using proper parallelometer mandrel.

### 2.2. PFM Crowns with Attachment and Metal Framework Casting Trial

**Figure 3:**
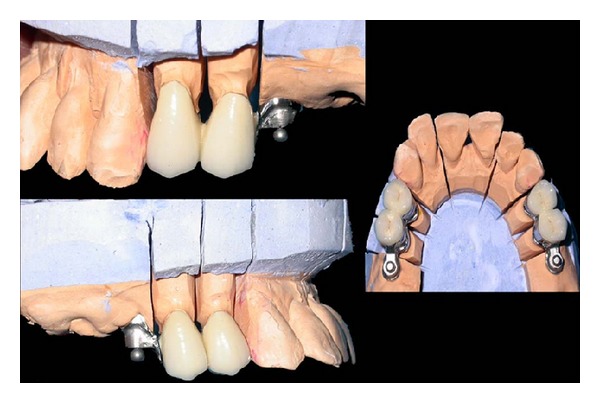
Joint PFM crown with attachment on the model.

**Figure 4:**
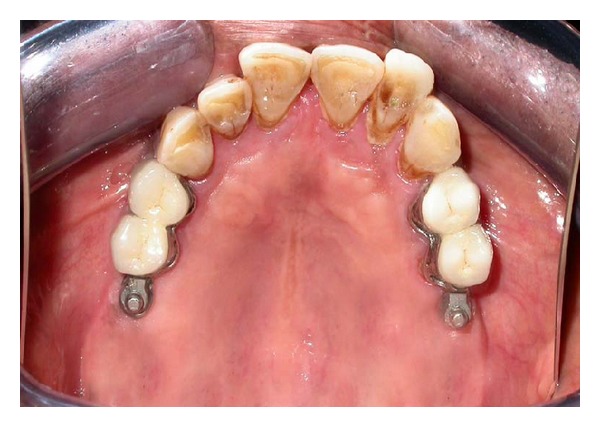
Try-in of the joint PFM crown with attachment.

Metal ceramic crowns waxed up with attachment structure were casted and porcelain firing was done. Joint crowns were fabricated with the attachments in the laboratory and the trial of the same was done to check the exact fit of the crowns (Figures 3 and 4).

**Figure 5:**
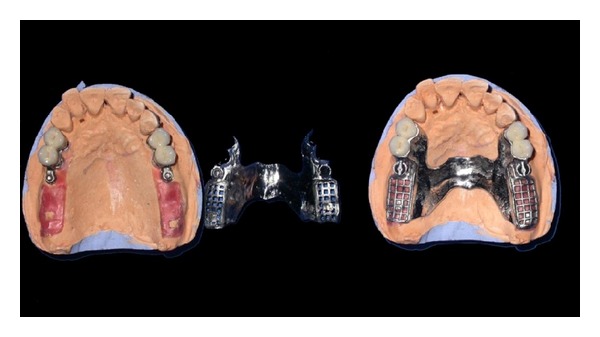
Metal framework on the model.

**Figure 6:**
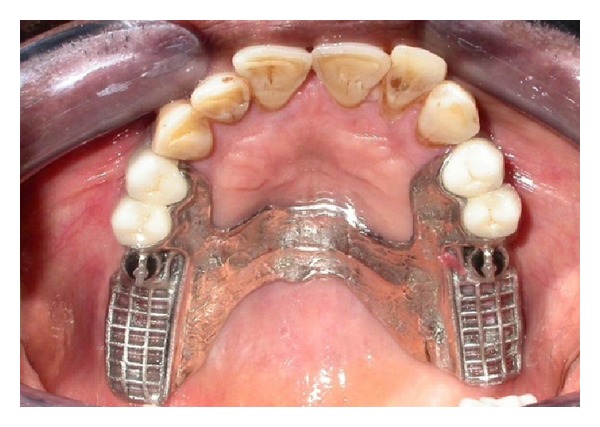
Framework trial in the patient's mouth.

Cast partial denture with attachment was fabricated in the laboratory and the metal framework trial was done in the patient's mouth for the accuracy of fit (Figures 5 and 6). Cast structure framework was checked up for stability and precision and jaw relation were recorded.

### 2.3. Wax-Up Trial

**Figure 7:**
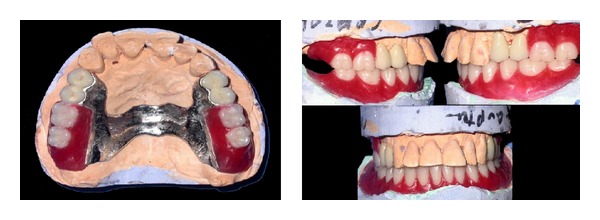
Waxed-up denture on model.

**Figure 8:**
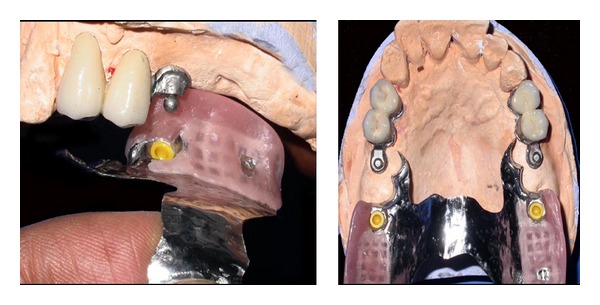
Acrylized prosthesis showing male and female O-ring attachment.

Waxing up of teeth was performed and teeth setting trial was done in patient's mouth (Figure 7). The trial denture was sent for acrylization and cast partial denture finished (Figure 8).

### 2.4. Positioning the Combined Prosthesis in the Patient's Mouth

**Figure 9:**
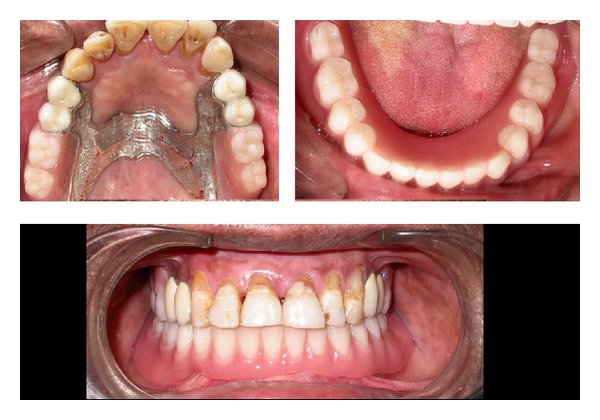
Postinsertion photo with combined prosthesis seated.

Trial seating of the finished prosthesis was performed and cementation of crowns was done using Glass Ionomer cement (GC Fuji). Attachments are protected with a thin layer of petroleum jelly (Vaseline) in order to easily remove cast partial denture after joint PFM crowns with attachment have been seated. Complete seating of finished maxillary combined prosthesis with extracoronal castable distal extension precision attachment was evaluated clinically and mandibular complete denture was also seated in the patient's mouth (Figure 9) and the patient was recalled after 24 hrs for postinsertion checkup.

## 3. Discussion 

Precision attachment is a connector consisting of two or more parts. One part is connected to a root, tooth, or implant and the other part to the prosthesis providing a mechanical connection between the two. These attachments allowed prosthesis to combine the advantage of fixed and removable restorations [5]. It was Dr. Herman Chayes who first reported the invention of attachment in the early 20th century [6]. Precision attachment gives a removable prosthesis the exceptional feature of improved esthetics, less postoperative adjustments, and improved comfort. It is mostly indicated for long-span edentulous arches, distal extension bases, and nonparallel abutments [7]. There is a wide range of attachments available for use in all manners of restorative procedures, from partial dentures to implant-supported prosthesis. By analyzing study models and X-rays, the clinician can make several important points of determination, each of which will influence final attachment selection. Apart from improving esthetics and retention of removable partial dentures, the availability of precision attachment has made designing of removable partial dentures more flexible. Various cases with esthetic and retention challenges can be solved with correct selection of attachment. Thus, unnecessary surgery and cutting of sound tooth for abutment preparation can be avoided in restoring missing teeth. However, precision attachments are not without disadvantages. Most of the attachments are very small and come with many parts to assemble. Construction of such attachment require skill from dental technicians which cannot be acquired easily and needs training. The parts of the attachment are usually exposed to wear and tear and needed to be replaced over time [8].

**Figure 10:**
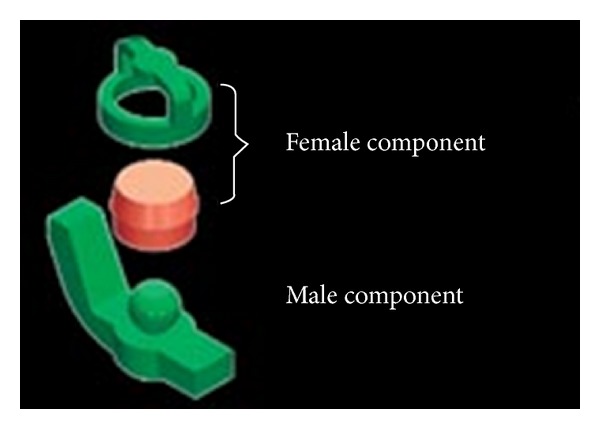
The RHEIN 83 OT CAP attachments system.

The RHEIN 83 OT CAP attachments system used in the case discussed in this paper is extracoronal castable attachment positioned on the distal of the crowns as an extension allowing a lot of vertical space for optimal aesthetics. The castable OT CAP male can be easily shaped together with the crowns during waxing-up stage avoiding complicated adaptation procedures like welding a metal attachment after crown casting. The male component design is sphere with a flat head and female component is retentive nylon caps which are color-coded according to different retentive properties (Figure 10).

## 4. Conclusion 

Removable partial dentures still have a good place as a treatment option for partially edentulous Kennedy's class I and class II conditions. With proper case selection and treatment plan, precision attachment such as RHEIN 83 OT CAP attachments system can be used to improve retention, esthetics, and function of removable partial denture. The above mentioned procedure using allows fabrication of very functional and comfortable prosthetic solution for the edentulous bilateral distal extension patient cases. Attachments retention can be monitored and upgraded during time just replacing retentive caps into the framework of dentures for patients comfort and satisfaction.
